# Colonel Bruce Vaughan Has a Good Time

**Published:** 1917-08-18

**Authors:** 


### KING EDWARD VII.'S HOSPITAL, CARDIFF.

### Colonel Bruce Vaughan has a Good Time.

Colonel Bruce Vaughan had a very pleasant day at the last meeting of the board of management on the 8th inst., for his main themes were the acknowledgment of gifts of money. He properly emphasised the importance of the service rendered to the nation by the Mercantile Marine, to which Messrs. W. R. Smith and Sons' one thousand guinea donation bore appreciative testimony. This handsome contribution was sent to Sir William James Thomas together with a donation of £450 for the Netley Hospital. "The reason Messrs. Smith and Sons state for making their generous contribution," Colonel Bruce Vaughan declared, "is a text from which I would like to preach for the whole afternoon, for does it not exactly state in plain, simple terms the reason why so many firms have also so generously endowed beds during the war in our hospital?" He appreciated the patriotic motive which prompted Messrs. Smith and Sons to make it, for he could appreciate as they had done the great and inestimable services rendered to the nation by the Mercantile Marine during the war. "The practical tribute to the gallantry and patriotism of their staff," he continued, "and the sailors in their employ, will be valued by everyone. It will be appreciated by men of all arms now serving at the Front, and by the sailors who are daily and hourly facing incalculable dangers at sea. It is but just that those of us who stay at home should make sacrifices also, and express in deeds and in adequate terms our appreciation of the gallantry of those who conduct our maritime operations. It will serve to remind them also that Messrs. W. R. Smith and Sons, and, thank God, many other firms as well, are not unmindful of the obligation cast upon them by the sacrifices that are being made by their employees in this war, and that the suffering thrown upon the weak and young, the women and children by the loss of their bread-winners, will in some slight measure be relieved and provided for in a hospital like ours."

Colonel Bruce Vaughan expressed the hope that Messrs. Smith and Sons' action may create the same desire in the minds and hearts of other shipowners to follow their example by paying homage to the British seamen, upon whose endurance and self-sacrifice to-day depends the safety of Great Britain, and, indeed, the very existence of the Empire.

### The New Convalescent Home.

Mr. Stevens, the *Dorothy*, had given £25 towards the furnishing, Sir William James Thomas had sent a contribution of £50, and Major-General Lee one of £25, to be continued annually, to this Home. There were now in the Home thirteen patients. There was already provision for seventeen, and it was hoped before the week was out there would be accommodation for another six.

### Relative Claims of R.A.M.C. and Civilian Doctors.

Colonel Vaughan drew the notice of the board to the fact that Major David Davies had called the attention of the Premier to the necessity of inquiring into the relative claims of the R.A.M.C. and civilian doctors, and said that Sir Garrod Thomas, speaking on the same question, had mentioned that many hospitals were largely staffed with unqualified men. In the Cardiff Hospital the resident staff had not been so efficient as it should be. He suggested that as far as the resident staffs of the great hospitals of the country were concerned, the War Office should delegate a sufficient number of newly-qualified men to act as residents, each for a period of six months. This hospital training would do much to fit them for their duties in the field, and would at the same time add greatly to the efficiency of the general hospitals. The present difficult and haphazard method of staffing the important voluntary hospitals in the kingdom was a scandal, and ought to be inquired into and a remedy found without delay. At the Chairman's suggestion the board authorised the secretary to write to Major David Davies saying that they sympathised with him in his demand for an inquiry.

### Gifts to the Hospital.

The following gifts were announced: £100 from Miss Emily C. Talbot towards the expenses of the *fête* to be held on the 23rd inst.; £1,000 from Mrs. Brockett Grover towards the building fund; £105 from Mr. Frederick Jones (Messrs. Frederick Jones and Co., shipowners, Cardiff) towards the building fund; 1,000 guineas from Messrs. W. R. Smith and Sons, shipowners, Cardiff, for the endowment of a bed in appreciation of the work done by their clerical staff and members of the crews of their steamers during the war; £10 from an anonymous donor for church furniture; £10 from an anonymous donor for the nurses' library.

